# Effects of color-flavor association on visual search process for reference pictures on beverage packaging: behavioral, electrophysiological, and causal mechanisms

**DOI:** 10.3389/fpsyg.2024.1433277

**Published:** 2024-09-09

**Authors:** Chen Cai, Le Zhang, Zitao Guo, Xin Fang, Zihan Quan

**Affiliations:** ^1^Department of Psychology, Normal College, Qingdao University, Qingdao, Shandong, China; ^2^School of Psychology, Center for Studies of Psychological Application, South China Normal University, Guangzhou, Guangdong, China

**Keywords:** color expectation, association strength, visual processing, perceptual representation, semantic representation, inferior temporal gyrus

## Abstract

The visual search for product packaging involves intricate cognitive processes that are prominently impacted by learned associations derived from extensive long-term experiences. The present research employed EEG technology and manipulated the color display of reference pictures on beverage bottles to explore the underlying neurocognitive pathways. Specifically, we aimed to investigate the influence of color-flavor association strength on the visual processing of such stimuli as well as the in-depth neural mechanisms. The behavioral results revealed that stimuli with strong association strength triggered the fastest response and the highest accuracy, compared with the stimuli with weak association strength and the achromatic ones. The EEG findings further substantiated that the chromatic stimuli evoked a more pronounced N2 component than achromatic ones, and the stimuli with strong association strength elicited larger P3 and smaller N400 amplitudes than the ones with weak association strength. Additionally, the source localization using sLORETA showed significant activations in the inferior temporal gyrus. In conclusion, our research suggests that (1) color expectations would guide visual search process and trigger faster responses to congruent visual stimuli, (2) both the initial perceptual representation and subsequent semantic representation play pivotal roles in effective visual search for the targets, and (3) the color-flavor association strength potentially exerts an impact on visual processing by modulating memory accessibility.

## 1 Introduction

The phenomenon where information from multiple sensory modalities collectively influences the perception of a stimulus is known as crossmodal correspondence (Spence, 2011). It is closely related to people’s life experience, at least in part from the results of statistical learning about the co-occurrence of stimuli pairings in the environment (Meng et al., 2023; Parise et al., 2014). A body of work has scrutinized the color-flavor incongruency effect during visual processing, demonstrating that the color-flavor congruent labels or packaging bolster the search efficiency of food, beverage or other products (Huang et al., 2021; Huang et al., 2019; Piqueras-Fiszman and Spence, 2011; Spence and Velasco, 2018; Velasco et al., 2015; Wan et al., 2022). Specifically, when purchasing food items with certain flavor (e.g., tomato-flavored potato chips), consumers are prone to engage in a color-based search for the target (e.g., searching for red packaging) unless the encountered color was incongruent with the expected ones, wherein they would shift to a word-based search strategy (Huang et al., 2021, 2019; Huang and Wan, 2019; Velasco et al., 2015). It suggests that the pre-presented flavor cues can prime individuals’ color expectation, directing their visual preferences in subsequent searching process.

Many studies have demonstrated the implicit and explicit associations between colors and product characteristics, marketing and psychological functioning, highlighting the pivotal role of color in social approach or avoidance behaviors (Bortolotti et al., 2023; Elliot and Maier, 2012; Singh and Srivastava, 2011). However, there exists a notable gap in the literature concerning the cognitive mechanisms underlying the visual search process for reference pictures on the packaging. Prominently featured on the packaging, particularly in the case of beverages, images are commonly taken as a key element of the packaging’s visual appearance. They serve not only to effectively capture consumers’ attention, but also to imperceptibly affect their purchasing behavior (Machiels et al., 2019; Simmonds and Spence, 2016). The flavor label cues can simultaneously activate the cognitive representation of the associated object, including information about the appearance of the stimulus (such as color, shape), the linguistic label, and its meaning (Smith and Magee, 1980). However, Piqueras-Fiszman et al. (2013) proposed that the influence of product images on individual’s behavioral intents surpassed text information. This suggests a preferential processing superiority for images over words when search for the products, indicating that individuals tend to be more sensitive to reference pictures compared to label words.

According to *the color diagnosticity hypothesis*, color was more likely to affect the recognition of objects with high color diagnosticity (HCD) rather than those with low color diagnosticity (LCD) (Tanaka and Presnell, 1999). They found that participants exhibited slower identification speed for achromatic and incongruent color versions of HCD objects relative to congruent color versions, which was not observed for LCD objects. This implied that color plays a critical role in the recognition of objects with stronger color associations. Furthermore, *the “Shape + Surface” model* proposed by Tanaka et al. (2001) highlights the significance of both perceptual and conceptual colors in HCD object recognition. For a HCD object, the association between its shape and characteristic color established based on the individual’s prior experiences triggers the mental representation and processing of the object in a top-down way (Dzulkifli and Mustafar, 2013; Tanaka et al., 2001; Zhou et al., 2021). Building upon our daily experiences, the present study selected five prevalent flavors of fruit beverages and their typical fruit images with typical colors (HCD). Moreover, with divergent co-occurrence frequencies of color-object pairings from the angle of social learning, we parted the color-flavor congruent condition into strong and weak associations to investigate their possible influence on visual search.

*Hypothesis I*: It is hypothesized that the responses to the pictures with color-flavor strong association would be faster and more accurate than to the ones with color-flavor weak association or the achromatic ones.

As revealed in previous research, the cueing word can automatically initiate associative processes, evoking a corresponding visual mental image in participants (Borst et al., 2011; Noorman et al., 2018). According to *the “Shape + Surface” model* of object recognition, the object’s name, such as a flavor label, is known to elicit its associated verbal and visual knowledge originated from daily experiences (Tanaka et al., 2001). Moreover, *the attention switch to memory (ASM) model* asserts that the attentional switch from the object’s external information to its relevant internal memory is an indispensable and inevitable process in visual search (Servais et al., 2023). When the mental representation of a flavor label is congruent with its memory storage, it would spontaneously capture focused attention and thereby facilitate cognitive processing (Han, 2018; Jung et al., 2019). This activation of long-term memory entails a dynamic interplay between episodic and semantic memory systems, with the former being linked to personal experiences and the latter to general knowledge (De Brigard et al., 2022; Greenberg and Verfaellie, 2010). Therefore, the mental representations, both visual and semantic, are instrumental in fostering a comprehensive recognition of the object (Collins and Olson, 2014; Davis et al., 2021).

Previous electrophysiological studies have established that the N2 component is related to early perceptual processing involved in attentional processes. This component can be further differentiated into three main subcomponents including an anterior N2 indexing perceptual deviance and conflict monitoring, a second anterior N2 predominantly signifying conflict control, and a posterior N2 reflecting target detection pertaining to visual attention (Bocquillon et al., 2014; Folstein and Van Petten, 2008; Tarbi et al., 2010; Żochowska and Nowicka, 2024). Given the indicative role of N2 component in general altering system (Liu et al., 2020; Suwazono et al., 2000), we assumed that participants would initially be in a state of heightened alertness when encountered the chromatic stimuli owing to color expectations, which is expected to manifest as increased N2 amplitudes than achromatic ones. In addition, the P3 amplitude has been taken as another indicator of attention allocation, with reduced amplitudes flagging limited cognitive resource dedicated to the task at hand (Beauducel et al., 2006; Kok, 1997; Leue and Beauducel, 2019; Polich, 2007). Consequently, it is anticipated that larger P3 amplitudes would be noted in trials that participants are actively engaged in and efficiently deploy attention (stimuli with strong association color), compared to trials that participants find difficult to handle (stimuli with weak association color).

Furthermore, the N400 component is intimately associated with semantic processing, in which semantic anomalies produce larger N400 amplitudes (Brouwer et al., 2016; Kutas and Federmeier, 2011; Kutas and Hillyard, 1980). A substantial body of research has extended its scope beyond linguistic materials to include non-linguistic stimuli such as pictures, which are used to characterize a modal and concrete conceptual systems (Khateb et al., 2010; Nigam et al., 1992). The N400 amplitude is also proved to be sensitive to error-monitoring and expectancy violation in that deviations from long-term memory representations lead to the onset of N400, implying a mismatch detection process (Dini et al., 2022; Misersky et al., 2021; Kutas and Federmeier, 2011). According to *the predictive coding framework*, the brain is an active, predictive organ that constantly generates predictions about the sensory input it is about to receive and updates these predictions based on incoming information (Clark, 2013; Friston, 2010; Rao and Ballard, 1999). The free-energy principle, a core concept of this framework, posits that the brain strives to minimize predictive error to achieve a stable state (Friston, 2003, 2009, 2010). We postulated that stimuli with weak association colors would generate a certain degree of prediction error due to their deviations from the typical expectations based on personal experiences and social learning, which could probably be manifested as prominent N400 amplitudes.

*Hypothesis II*: There would be larger N2 and P3 amplitudes elicited by the strong association color condition owing to effective attention allocation, while stimuli in the weak association color condition would elicit more negative N400 amplitudes due to greater violation of expectation.

Extensive behavioral and electrophysiological studies have demonstrated that object visual search processes encompass the generation and manipulation of visual mental imagery, as well as the engagement of semantic representations (Cao et al., 2018; Collins and Olson, 2014; Moriya, 2018). Visual mental imagery pertains to the internal evocation of an object’s perceptual attributes and semantic representation involves the cognitive construction of an object’s conceptual knowledge. The color-flavor association strength is defined by the extent to which a stimulus matches its stored representation (i.e., template) in memory. Prior neuroimaging research has unanimously exhibited the crucial role of temporal lobe in object processing, which is implicated in visual perception and memory (Hamamé et al., 2012; Miyashita, 1993; Tong et al., 2024; Ramezanpour and Fallah, 2022). Miyashita (1993) suggested that inferior temporal cortex is critical for the formation of visual long-term memory and for the interplay between perception and memory. Tanaka et al. (2001) also proposed that the left inferior temporal area is more involved with invoking knowledge about the inherent colors rather than lexical information of objects. Our study aimed to elucidate how the color-flavor association strength modulates visual search processes and attempted to localize the related brain regions by analyzing the neural source of ERP difference waves between conditions with strong and weak association colors.

*Hypothesis III*: The color-flavor association strength had an impact on attentional processes, potentially eliciting specific neural responses that were supposed to be mainly represented in the activation of temporal lobe, a brain region involved in high-level visual processing.

## 2 Methods

### 2.1 Participants

*A priori* power analysis conducted by G*Power 3.1.9.7 (Faul et al., 2007) exhibited that a sample size of 28 is required to reach 80% power for the present one-factor within-subject design, with a medium effect size of 0.25 and an alpha of 0.05. A total of 41 undergraduates (19.27 ± 1.56 years; 13 males) from Qingdao University were recruited in this study for possible data loss or exclusion. Two participants were excluded from the data pool afterward due to frequent head movements during the EEG recording, resulting in 39 participants included in subsequent analyses. All participants had normal or corrected-to-normal vision without color blindness or any neurological history. Written informed consent was obtained from each participant, and the experiments were approved by the Institutional Review Board of Psychology, Qingdao University on March 21, 2022 (*IRB No. QDU202203210001*).

### 2.2 Tasks and materials

We used E-Prime 3.0 software^1^ to present visual stimuli and record the behavioral data. Adobe Photoshop CS 6 software was utilized to depict beverage bottle images against a black background. Each flavor label (each subtending 5.72° horizontally and 1.34° vertically) was composed of three Chinese characters (boldface, font size 12). Four images of the beverage bottle (each subtending 5.91° horizontally and 9.72° vertically) simultaneously presented in the center of four quadrants of the screen, with a resolution of 80 × 150 pixels. These visual stimuli were presented on a 23.8-inch monitor with a resolution of 1,600 × 900 pixels and a refresh rate of 60 Hz. The viewing distance for the participants was approximately 60 cm.

A preliminary survey comprised of two questionnaires were conducted to screen the specific colors of reference pictures that correspond to colors in the conditions with strong and weak association strength. The survey was posted on the WJX online questionnaire platform (Changsha Ranxing IT LTD., Changsha, Hunan, China). Both questionnaires required participants to choose one color that they perceived as the best match for each given flavor label. The colors that were selected most and least frequently were, separately, designated as the strong and weak association colors for the very reference pictures. Five common fruit flavors, each associated with at least two colors, were chosen for the study: lemon, peach, orange, grape, and mango. The color options included pink (R: 255, G: 105, B: 185), purple (R: 151, G: 2, B: 178), yellow (R: 251, G: 237, B: 6), orange (R: 255, G: 165, B: 0), yellow ochre (R: 250, G: 202, B: 94), yellow green (R: 154; G: 205, B: 50), green (R: 0; G: 128, B: 0), spring green (R: 0, G: 142, B: 87), and lime green (R: 50, G: 205, B: 50) in Questionnaire 1, which was used to differentiate between strong and weak association colors for specific flavor labels. To refine the color options and prevent overlap, Questionnaire 2 incorporated additional colors of gold (R: 255, G: 215, B: 0) and green yellow (R: 173, G: 255, B: 47), ensuring that strong and weak association colors remained distinct and did not overlap across different flavors. As a result, Questionnaire 2 presented four yellowish colors (yellow, orange, yellow ochre, and gold) or five greenish colors (yellow green, green, spring green, lime green, and green yellow) for strong and weak association conditions, respectively. The ultimate designation of reference pictures in the condition with strong association strength comprised peach in pink color, grape in purple color, lemon in yellow color, orange in orange color, and mango in yellow ochre color. Those in the condition with weak association strength consist of peach in gold color, grape in green color, lemon in spring green color, orange in lime green color, and mango in green yellow color. Reference pictures in the achromatic color condition were presented in gray colors (R: 135; G: 135; B: 135).

### 2.3 Design

**Figure 1:**
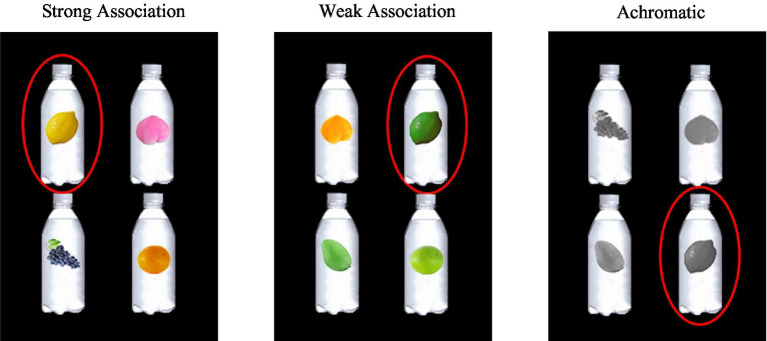
The experimental design with three conditions. Taking “柠檬味” (lemon flavor) as an example of the target flavor label to illustrate the specific experimental design under the three conditions. From left to right shows the strong association color, weak association color, and achromatic color conditions, and the images circled in the red circles were the targets that participants should select under these conditions.

A one-factor (color display: color-flavor strong association color, color-flavor weak association color, and achromatic color) within-subject design was applied and the sample illustrations in three experimental conditions are presented in Figure 1.

### 2.4 Procedure

**Figure 2:**
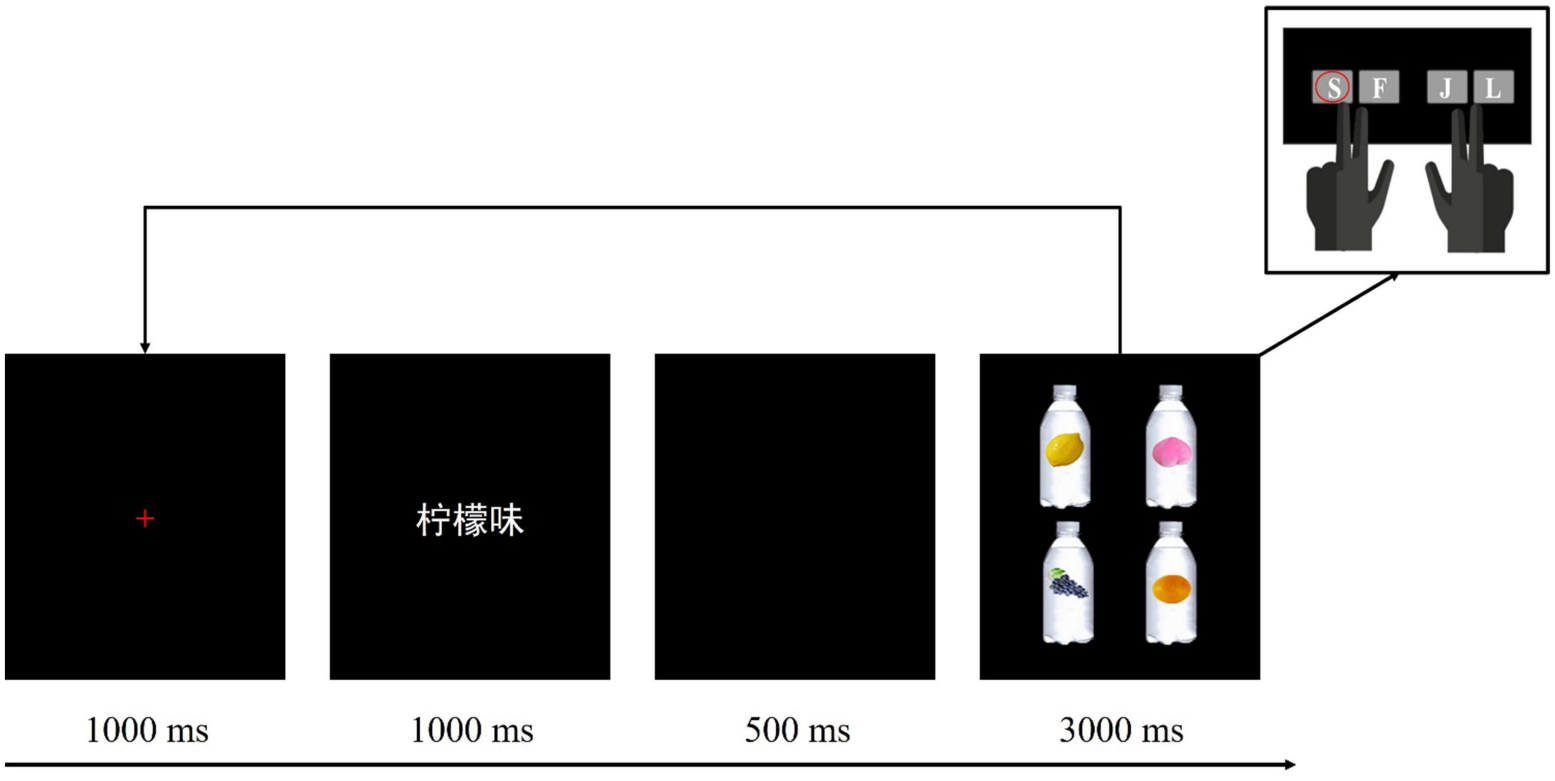
The experimental procedure in three conditions. Taking “柠檬味” (lemon flavor) in the strong association color condition as an example to illustrate that the participants should use their left middle finger to press “S” key on the computer keyboard when the target was presented in the upper left quadrant.

Prior to the experiment, the participants first signed a consent sheet addressing the matters they needed to be aware of. After that, they were guided to go through the preparatory work for the EEG experiment and then were instructed to engage in the formal task. As illustrated in Figure 2, each trial in the experiment started with a 1,000 ms red cross in the center of the screen, then a flavor label word consisting of three Chinese characters presented for 1,000 ms, after a 500 ms blank screen, four beverage bottle images were presented in the four quadrants of the screen for 3,000 ms. The participants were instructed to search for the target bottle image labeled with corresponding reference picture by using the index or middle finger to press designated keys on the keyboard. The practice block contained 15 trials, with five trials for each experimental condition, and four beverage bottle images with different reference pictures were randomly presented in the four quadrants at each time. The formal experiment contained 300 trials, evenly divided into three blocks. The order of trials and conditions within each block was randomized for each participant. It took approximately 35 min to finish all the task.

### 2.5 Electrophysiological (EEG) data recording and preprocessing

The EEG signals were recorded from 64 Ag/AgCl active electrodes embedded in a conductive cap (actiCHamp, Brain Products, Gilching, Germany) conforming to the 10–20 international EEG system. The impedances were kept below 5 KΩ, with electrooculogram (EOG) signals recorded with Fp1 and Cz serving as the online reference. Signals were digitized at a 1,000 Hz sampling rate, amplified by a Brain Product actiCHamp amplifier system with a 0.01–100 Hz band-pass filter, and recorded by the Brain Vision Recorder 2.1 software (Brain Products, Gilching, Germany). All channels were filtered with a range of 0.05–30 Hz and re-referenced offline to an average of bilateral mastoids. Data analysis was performed using Brain Vision Analyzer 2.1 software. We excluded the trials that were contaminated by positive and negative deflections that exceeded ±200 μV. The epochs were generated between −200 ms pre-stimulus onset and 1,200 ms post-stimulus onset, with −200 to 0 ms serving as a baseline. Only ERPs with correct response were averaged under each color display condition.

### 2.6 ERP data analyses

Based on previous studies (Cao et al., 2018; Dini et al., 2022; Nigam et al., 1992) and the grand average waveforms for each electrode site, the mean amplitudes of the N2, P3, and N400 components in the present study were examined over 160–240 ms, 300–400 ms, and 400–500 ms, respectively. Frontal (F3, Fz, and F4), central (C3, Cz, and C4), and parietal (P3, Pz, and P4) electrodes were included in the statistical analysis. A 3 (color display: color-flavor strong association color, color-flavor weak association color, and achromatic color) × 3 (brain region: frontal, central, and parietal) repeated-measure analysis of variance (ANOVA) was performed in each time window using IBM SPSS Statistics (Version 27.0). The *p*-values were reported after the Greenhouse–Geisser correction when the sphericity assumption was not satisfied and the default Fisher’s least significant difference (LSD) method was used for multiple comparisons.

The Brainstorm software^2^, which operates on the MATLAB platform, was used for ERPs source analysis. Firstly, without recording any structural MRI ourselves, we adopted the default ICBM152 template established by the International Consortium for Brain Mapping (ICBM) and applied it for the subsequent process of head modeling correction. Then, we used the default structural data to process our EEG data, which had been preprocessed using Brain Vision Analyzer 2.1 software. For the database importation, EEG signals ranging from −200 to 1,200 ms were extracted, with the interval from −200 to 0 ms as a baseline. The procedure continued with the alignment of electrodes and magnetic resonance imaging (MRI) for accurate head model computation. Next, we implemented the “no noise modeling matrix” to compute the noise covariance matrix. Last, the sLORETA (standardized Low Resolution Electromagnetic Tomography) method was adopted to explore the neural source of difference waves within the specific time windows of 300–400 ms and 400–500 ms, aiming to unravel the neural mechanisms underlying color-flavor association strength during the visual search process.

## 3 Results

### 3.1 Behavioral results

Within the correct trials (93.778%), we excluded response times (RTs) which were shorter than 200 ms or three standard deviations (2559.581 ms) longer than the group means from the following data analysis, resulting in 1.139% data being excluded. Similarly, when the sphericity assumption was not satisfied, the *p*-values were calculated with the Greenhouse–Geisser correction and the Fisher’s LSD method was used for multiple comparisons.

A repeated-measure ANOVA analysis was performed and the results revealed a significant main effect of color display both on RTs, *F*(2, 76) = 73.186, *p* < 0.001, $\eta_{p}^{2}$ = 0.658, and on ACCs, *F*(1.486, 56.467) = 5.855, *p =* 0.010, $\eta_{p}^{2}$ = 0.134. Multiple comparisons showed that participants responded slower and less accurately to the weak association color than to the strong association color (RT: *p* < 0.001, 95% CI = [134.440, 192.046]; ACC: *p* = 0.003, 95% CI = [−0.041, −0.009]) and also to the achromatic color (RT: *p* < 0.001, 95% CI = [85.454, 136.321]; ACC: *p* = 0.018, 95% CI = [−0.051, −0.005]). Furthermore, participants responded much slower to the achromatic color than to the strong association color, *p* < 0.001, 95% CI = [23.067, 81.643], but not less accurately, *p* = 0.670, 95% CI = [−0.011, 0.018]. The behavioral results can be seen in Table 1.

**Table 1:** Results of repeated measures ANOVA on RTs (ms) and ACCs in different color conditions.

	Color display	*M ± SD*	*F*	*p*	$\eta_{p}^{2}$
RT	Strong association	1040.491 ± 162.936	73.186	<0.001	0.658
	Weak association	1203.734 ± 182.789			
	Achromatic	1092.846 ± 181.371			
ACC	Strong association	0.945 ± 0.050	5.855	0.010	0.134
	Weak association	0.920 ± 0.079			
	Achromatic	0.948 ± 0.036			

RT, response time; ACC, accuracy.

### 3.2 ERP results

#### 3.2.1 Component analysis

A significant main effect of color display was found on N2 component, *F*(1.594, 60.574) = 9.003, *p* < 0.001, $\eta_{p}^{2}$ = 0.192. Pairwise comparisons showed both the N2 amplitudes of strong and weak association color were more negative than those of achromatic color (strong association vs. achromatic: *p* = 0.002, 95% CI = [−0.832, −0.204]; weak association vs. achromatic: *p* = 0.002, 95% CI = [−0.972, −0.230]). Whereas no such significant differences were detected between the N2 amplitudes of strong association color and those of weak association color, *p* = 0.472, 95% CI = [−0.314, 0.148]. Moreover, the main effect of brain region was significant, *F*(1.122, 42.652) = 22.719, *p* < 0.001, $\eta_{p}^{2}$ = 0.374, with a more negative frontal N2 than central N2, *p* = 0.004, 95% CI = [−0.893, −0.177], and a more negative central N2 than parietal N2, *p* < 0.001, 95% CI = [−2.429, −1.033]. The interaction effect between color display and brain region was not significant, *F*(2.095, 79.628) = 1.006, *p* = 0.373, $\eta_{p}^{2}$ = 0.026.

As for the P3 component, the main effect of color display was not significant, *F*(2, 76) = 2.446, *p* = 0.093, $\eta_{p}^{2}$ = 0.060. However, a significant main effect of brain region was observed, *F*(1.157, 43.952) = 109.341, *p* < 0.001, $\eta_{p}^{2}$ = 0.742, suggesting that the parietal P3 was larger than the central P3, *p* < 0.001, 95% CI = [2.594, 3.848], and that the central P3 was larger than the frontal P3, *p* < 0.001, 95% CI = [1.406, 2.272]. There was also a significant color display × brain region interaction, *F*(2.256, 85.742) = 6.111, *p* = 0.002, $\eta_{p}^{2}$ = 0.139. The stratified analysis revealed that the strong association color displayed a more positive P3 than the weak association color both in the central and parietal regions (central: *p* = 0.037, 95% CI = [0.026, 0.756]; parietal: *p* < 0.001, 95% CI = [0.372, 1.072]). Furthermore, the strong association color also exhibited a more positive P3 than the achromatic color in the parietal region, *p* = 0.009, 95% CI = [0.164, 1.097]. However, no such differences were found between the weak association color and the achromatic color across all three brain regions, *p* = 0.272 ~ 0.671, as well as the pairwise comparisons of three color displays in the frontal region, *p* = 0.260 ~ 0.881.

**Figure 3:**
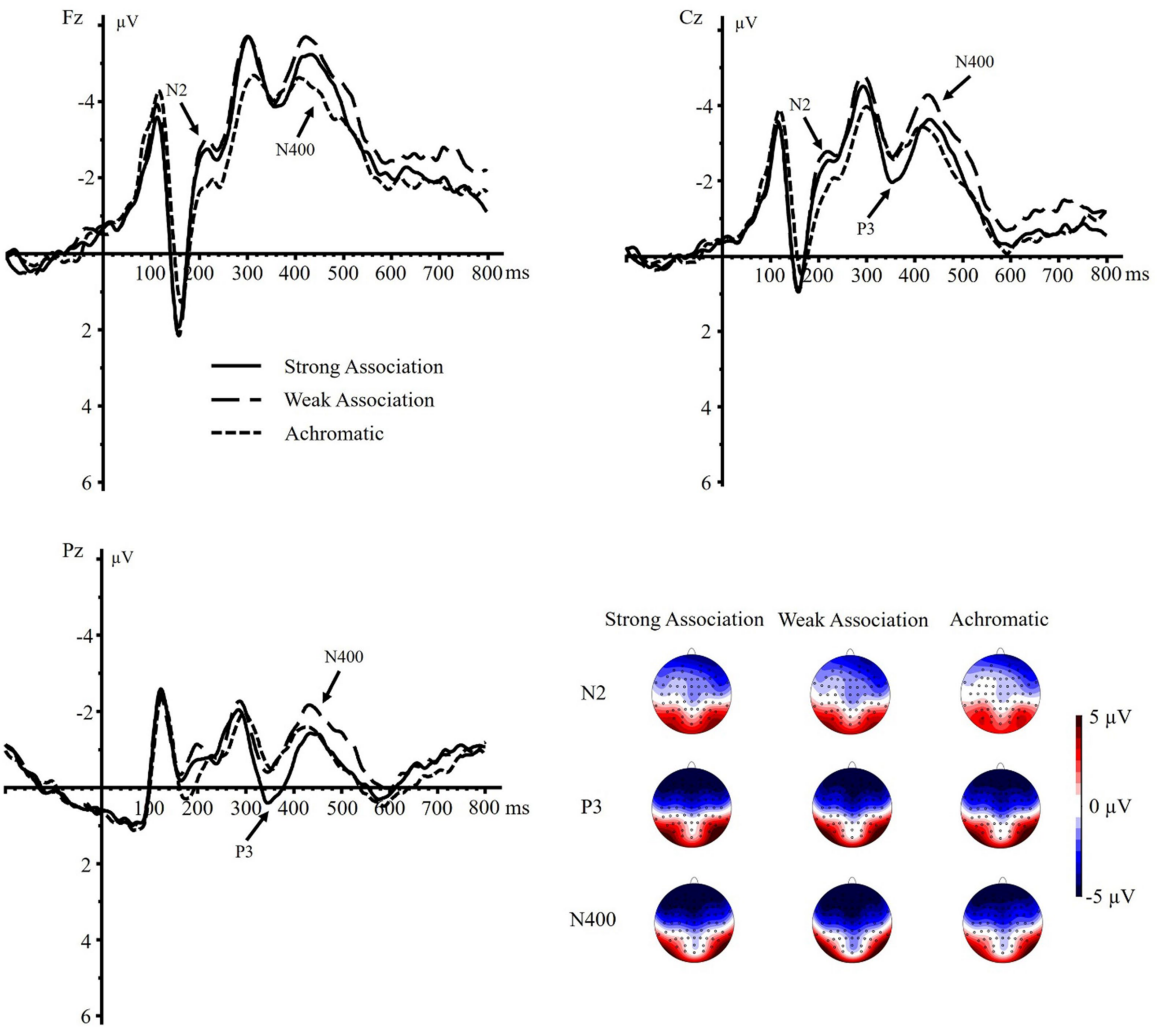
Grand-averaged ERPs and the topographic maps of N2 (160–240 ms), P3 (300–400 ms), and N400 (400–500 ms) elicited by different conditions on electrodes Fz, Cz, and Pz.

Regarding the N400 component, a significant main effect of color display was detected, *F*(1.730, 65.749) = 8.609, *p* < 0.001, $\eta_{p}^{2}$ = 0.185. The weak association color elicited a more negative N400 than the strong association color (*p* = 0.009, 95% CI = [−0.820, −0.124]) and achromatic color (*p* = 0.001, 95% CI = [−1.258, −0.335]), whereas no such difference was detected between the strong association and achromatic colors (*p* = 0.070, 95% CI = [−0.676, 0.028]). The main effect of brain region was also significant, *F*(1.126, 42.776) = 88.662, *p* < 0.001, $\eta_{p}^{2}$ = 0.700. The frontal N400 was larger than the central N400, *p* < 0.001, 95% CI = [−2.122, −1.283], and the central N400 was larger than the parietal N400, *p* < 0.001, 95% CI = [−3.089, −1.989]. Moreover, the color display × brain region interaction was significant, *F*(1.914, 72.722) = 3.793, *p* = 0.029, $\eta_{p}^{2}$ = 0.091. Further analysis showed that in the frontal region, both the strong and weak association color elicited a more negative N400 than the achromatic color (strong vs. achromatic: *p* = 0.003, 95% CI = [−1.005, −0.232]; weak vs. achromatic: *p* < 0.001, 95% CI = [−1.340, −0.379]), whereas no significant difference was found between the former two, *p* = 0.262, 95% CI = [−0.187, 0.670]. Additionally, both in the central and parietal regions, the weak association color triggered a more negative N400 amplitude than the strong association color (central: *p* = 0.009, 95% CI = [−0.871, −0.136]; parietal: *p* < 0.001, 95% CI = [−1.050, −0.293]) as well as the achromatic color (central: *p* = 0.001, 95% CI = [−1.386, −0.381]; parietal: *p* = 0.012, 95% CI = [−1.139, −0.153]). But no differences between the strong association and achromatic colors were revealed in these regions (central: *p* = 0.062, 95% CI = [−0.780, 0.021]; parietal: *p* = 0.916, 95% CI = [−0.518, 0.466]) (Figure 3).

#### 3.2.2 Source location analysis

**Figure 4:**
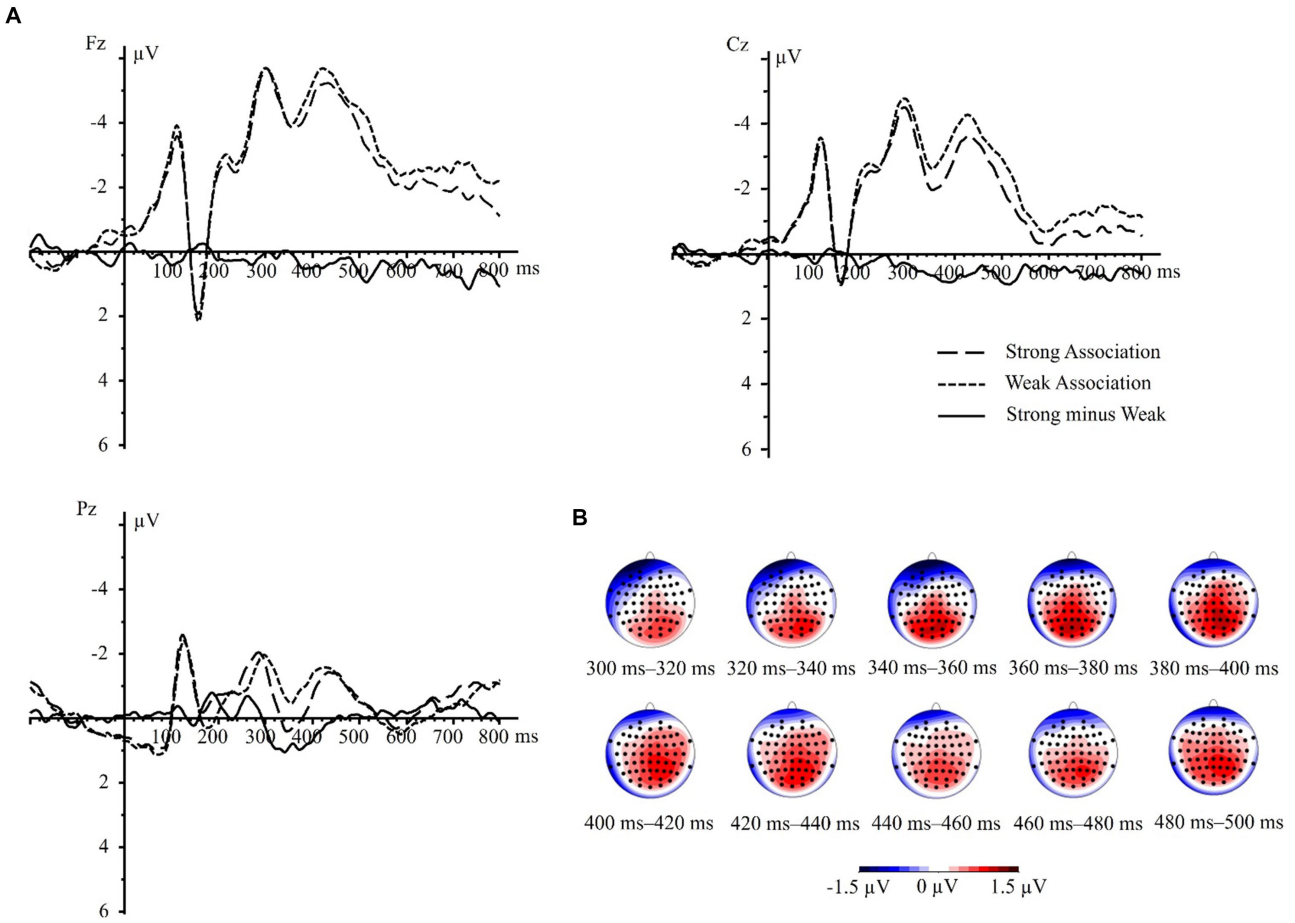
The difference waves (strong minus weak) and its topographic maps. **(A)** Strong association minus weak association difference waves in electrodes Fz, Cz, and Pz. **(B)** The topographic maps illustrating the mean difference in component amplitudes with time windows of 300–400 ms and 400–500 ms at interval of 20 ms.

In the present study, source location analysis was conducted using the sLORETA algorithm method on the difference waves between strong and weak association color conditions. As shown in Figures 4A,B, and as indicted by the results of ERP component analysis, the differential areas mainly located at central and posterior electrodes during the time windows of 300–400 ms and 400–500 ms. Consequently, we performed sLORETA analysis with the same nine electrodes targeting three brain regions, corresponding to the two time windows of 300–400 ms and 400–500 ms identified in the ERP component analysis.

**Figure 5:**
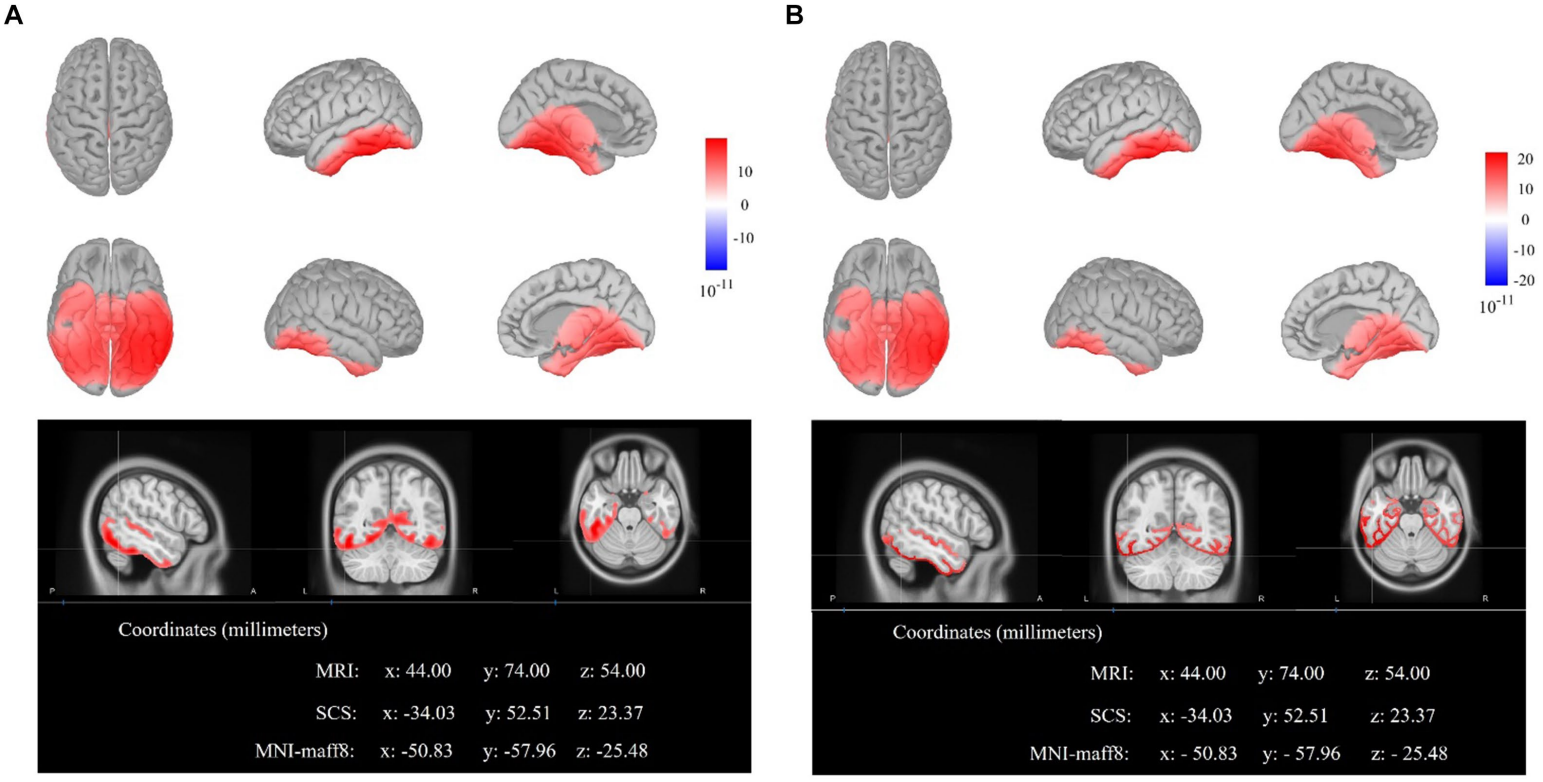
The electroencephalogram source locations of the difference waves (strong minus weak). **(A)** The sLORETA displays of activated neural source on cortex and MRI for the time window of 300–400 ms. **(B)** The sLORETA displays of activated neural source on cortex and MRI for the time window of 400–500 ms.

As illustrated in Figure 5, the results of sLORETA locating the neural sources of difference waves between strong association color and weak association color within the time window of 300–400 ms illustrated that the maximal activations emerged in the left inferior temporal gyrus (ITG) (MNI coordinates: *x* = −50.83, *y* = −57.96, *z* = −25.48). Furthermore, the results of sLORETA within 400–500 ms also showed the maximal activations in the left ITG (MNI coordinates: *x* = −50.83, *y* = −57.96, *z* = −25.48).

## 4 Discussion

The flavor information presented through flavor label word would activate individuals’ color expectation, which in turn facilitates the visual search for target packaging (Huang et al., 2019; Peng and Wan, 2022). In alignment with this view, our findings revealed that the search for stimuli with strong color-flavor associations was characterized by the most rapid response speed and highest accuracy among three color display conditions. This is probably attributed to the brain’s ability to form robust memory traces for stimuli with strong associations. With repeated exposure, our brain strengthens the neural connections associated with these pairings, making them more readily accessible and retrievable from memory (Betts et al., 2019; Higgins and Hayes, 2019). What is more, the observed performance decrements for stimuli with weak association colors compared to those achromatic ones unveiled that the former activated greater cognitive conflicts from, if any, perceptual representations and semantic representations. A well-integrated associative network built upon consistent perceptual and semantic experiences is vital to optimal task performance (Hung et al., 2020; Kvasova et al., 2023).

On the other hand, the achromatic stimuli were incapable of offering color information, which failed to meet the initial color expectations than ones with strong association colors, but circumvented the disruptive effects of insufficient chromatic information compared to the ones with weak association colors. It suggested that the participants might mainly trigger a color-based visual search initially, and then shifted to a shape-based search when the expected color was absent (Tanaka et al., 2001; Therriault et al., 2009). From the perspective of *the predictive coding framework* (Clark, 2013; Friston, 2003, 2009, 2010; Rao and Ballard, 1999), stimuli with achromatic and weak association colors both elicited predictive errors based on participants’ expectations, leaving them to adjust and optimize their predictions by minimizing free energy related to processing colors in order to better match actual sensory inputs. Because the uncommon or atypical color combinations displayed by stimuli with weak association are likely to increase the prediction errors, which prompted the brain to suppress the dominating associative colors and process the present ones, leading to a slower and more inefficient visual search. However, the achromatic stimuli directed participants’ attention to the correct targets entirely by the shape of reference pictures, which, in a way, decreased the degree of prediction errors invoked by the color information.

The EEG results suggested that both the conditions with strong and weak association color (i.e., the chromatic color conditions) elicited larger N2 amplitudes with a frontocentral scalp distribution than the achromatic condition. Given that prior studies convergently deemed the N2 component as a marker for attention processes, with enhanced anterior N2 amplitudes associated with the detection of perceptual novelty or mismatch from attended visual stimuli (Bocquillon et al., 2014; Folstein and Van Petten, 2008; Zheng et al., 2023), stimuli displayed in the condition with weak association color should have resulted in elevated N2 amplitudes due to greater deviances from long-term experiences. However, it is noteworthy that no significant differences of N2 amplitudes between the strong and weak association conditions were observed in our study. This might be attributed to that the visual stimuli trigger heightened general alertness status during early stage of attentional processing (Chu et al., 2019; Suwazono et al., 2000), since both conditions were nested with chromatic stimuli that are somewhat analogous to the contents originating from mental imagery. To go step further, the insignificant N2 differences may be mirrored in attentional allocation during the early stage of visual search, which is free from conflict monitoring but rather connected with attentional alertness. Our results showed that the color stimuli attracted more attentional alertness compared to grayscale, regardless of whether the color-flavor association strength was strong or weak.

In addition, Kok (2001) states that a lower degree of matching with the mental representations or templates leads to smaller P3 amplitudes. Likewise, the stimuli displayed in the strong association colors induced larger P3 amplitudes, compared to those with weak association colors and achromatic color, on the basis that it was strongly accordant with templates stored in memory. Hence, our findings corroborated the notion that the P3 component reflects attentional resource allocation, in which more positive P3 amplitudes are associated with lower cognitive load (Beauducel et al., 2006; Kok, 1997; Nisar et al., 2023). That is to say, reference pictures with strong color-flavor association necessitate less cognitive efforts on account of their consistency with color expectations, which further underlies the critical role of color information in visual search for HCD objects. Meanwhile, the more pronounced negative N400 amplitudes over central and parietal scalp evoked by the stimuli with weak association colors can be attributed to expectation violations (Ganis and Kutas, 2003; Kutas and Federmeier, 2011). Dini et al. (2022) has discussed the relationship between the N400 effect and the error-monitoring process, extending the application of the *predictive coding theory* to non-linguistic domains, such as brands and pictures. The results in our study suggest that the weakly associated colors caused deviances between the brain predictions and the actual sensory inputs, resulting in prediction errors that consume more cognitive efforts to optimize the brain’s internal model of the environment (Clark, 2013; Friston, 2010; Rao and Ballard, 1999). This also captures the idea of *the ASM model* that emphasizes the attentional translation from external environment to internal memory (Hautekiet et al., 2023). What is more, we broaden its application by highlighting the involvement not only of episodic memory but also of semantic memory in the context of our study.

However, we did not observe a significant difference in N400 amplitude between strong and weak association colors in the frontal region, which we thought could probably be traced back to the stimuli used in the present study. Unlike most previous research on images that reported a pronounced N400 effect over the frontal area (Ganis et al., 1996; Wu and Coulson, 2005), we applied a flavor label prime before fruit images associated with specific beverage flavors. Likewise, the study of Dini et al. (2022) that used brand logos found a pronounced N400 effect in the central region instead of the frontal region. Therefore, it is reasonable to suggest that the observed N400 effect may pertain to the cross-modal integration between linguistic modality (flavor label words) and visual modality (fruit images), as well as the modulation of attentional allocation to the image information. Moreover, the frontal lobe is known for its role in higher-level cognitive functions such as planning and executive control (Alvarez and Emory, 2006; Otero and Barker, 2014; Torralva et al., 2016), whereas the central and parietal regions are associated with visual perception and sensory integration (Dijkstra et al., 2017; Husain, 2016). Given this, the central-parietal distribution of the N400 effect in our task seems plausible, although further investigation is still needed.

Furthermore, the source localization of ERP difference waves displayed the maximum activation within the left ITG, which might be an unneglectable brain region related to high-level visual processing. Object recognition and memory retrieval have been closely linked to temporal lobe, particularly the ITG (Conway, 2018; Miller et al., 1991). An fMRI study by Ranganath et al. (2004) confirms that the ITG is involved in associative long-term memory retrieval and object representation, which also impacts the attentional direction during visual search tasks. The present study corroborated this functional role of the ITG, proving the effect of color-flavor association strength on participants’ visual search for targets is taken from the accessibility of their representations in memory. In conjunction with the aforementioned, the flavor label evoked participants’ color expectation based on preliminarily learned associations, and directed their attention in the way of comparing the perceptual information with the mental representations retrieved from long-term memory. Notably, the left ITG is also thought to be critical to semantic representation and is implicated in the meaning retrieval (Liuzzi et al., 2020; Whitney et al., 2010). Specifically, elevated activations observed in the left ITG flags the retrieval of object color knowledge, which elucidates that visual representations are indeed triggered by object-color associations (Tanaka et al., 2001). In summary, the findings in our research indicate that semantic processing is incorporated in the visual search process for reference pictures as well as illuminate that the left ITG contributes to visual information processing and plays an integral role in memory retrieval and associative processing.

To sum up, the results of our study underscored the integral role of color expectation in visual search for beverage pictures. This finding corroborated the view of *the color diagnosticity hypothesis* and *the “Shape + Surface” model* that color is integral to object recognition. Unlike prior research focused on recognizing congruent and incongruent objects, we innovatively incorporated the variable of color-flavor association strength under congruent scenario and explored the underlying possible multisensory mechanisms. In addition, the EEG results of our study, especially the N400 effect, might indicate the activation of semantic knowledge during visual search and thereby extend *the ASM model* from episodic memory to semantic memory retrieval. However, there are several limitations to the current research that warrant acknowledging. First of all, we only utilized sLORETA method for the source localization, which is relatively insufficient for a direct and thorough examination of brain activation. Drawing from previous literatures (Freud et al., 2016; Mahon and Almeida, 2024) and the EEG results in the present study, the dorsal visual pathway might be more important than the ventral one in object visual perception. Therefore, future research should consider employing techniques with higher spatial resolution, such as functional magnetic resonance imaging (fMRI), to delve into the intrinsic functional connectivity between various brain regions and make attempts to verify whether the former speculation holds true. Secondly, the materials utilized in this research were relatively simplistic, with association strength being manipulated solely by changes in the color of reference pictures. To better understand the role of association strength in object recognition, future research should involve more diversified and true-to-life forms of stimuli, such as fruit images and labels presenting together on the same packaging. Moreover, we recorded the ERP data with a 0.01–100 Hz online band-pass filter and pre-processed it with another 0.05–30 Hz offline filter, which, in some degree, might induce noises or artifacts in the data. Therefore, subsequent research might benefit from applying the band-pass filter only once to maintain data precision. Finally, it should be noted that the weak association condition may turn into a degree of “strong” association through the repetitive presentation method in this study. Accordingly, future studies could reduce the proportion of trials with weak association or consider applying a single-trial approach to explore the potential trial order effect.

## 5 Conclusion

The current research underscores the predominant role of color in the product search process, suggesting that the color expectations shaped by life experience guide the visual search efficiency for reference pictures on beverage bottles. More importantly, rooted in the EEG results, the presentation of a flavor label not only evokes the perceptual attributes associated with the corresponding fruit image but also activates its conceptual representations, reflecting the temporal course from lower-level perceptual processing to higher-level semantic integration. Collectively, our findings highlight the significance of the perceptual-semantic congruency in facilitating efficient cognitive processing, which in turn improves subsequent performance in visual search tasks. Furthermore, the pronounced activations observed in the left ITG through source location analysis of difference waves illuminates the interplay between memory retrieval and visual information processing. To sum up, our research paves the way of interpreting the crucial role of color-flavor association strength in visual search processes from both behavioral and electrophysiological perspectives.

## Data Availability

The original contributions presented in the study are included in the article/supplementary material, further inquiries can be directed to the corresponding author.

## Footnotes

^1^ https://pstnet.com/products/e-prime/

^2^ https://neuroimage.usc.edu/brainstorm/
